# The Antitumor Effect of TPD52L2 Silencing on Oxaliplatin-Resistant Gastric Carcinoma Is Related to Endoplasmic Reticulum Stress *In Vitro*

**DOI:** 10.1155/2022/4451178

**Published:** 2022-01-18

**Authors:** Yu Zhang, Dejun Yang, Ziran Wei, Xin Zhang, Zunqi Hu, Hongbing Fu, Jiapeng Xu, Weijun Wang

**Affiliations:** Department of Gastrointestinal Surgery, Second Affiliated Hospital of Naval Medical University, Shanghai 200003, China

## Abstract

Tumor protein D52-like 2 or simply TPD52L2 belongs to the TPD52 family which has been implicated in a variety of human carcinomas. However, the TPD52L2 function in the gastric carcinoma oxaliplatin (OXA) resistance remains elusive. The main objective of this study is to evaluate the TPD52L2 effect in OXA-resistant gastric carcinoma cells *in vitro*. Oxaliplatin-resistant gastric carcinoma cells were generated in MGC-803 and SGC-7901 cells. siRNA-mediated knockdown of TPD52L2 was investigated in OXA-resistant MGC-803-OXA and SGC-7901-OXA cells. qRT-PCR was performed to assess the expression level of TPD52L2 mRNA. TPD52L2 protein expression level, apoptosis, and endoplasmic reticulum (ER) stress-associated proteins were identified via immunoblotting analysis. MTT assay was conducted for the evaluation of cell viability, while colony-forming activity was carried out via crystal violet staining. SGC-7901-OXA and MGC-803-OXA cells were found to be more resistant to OXA, as compared to the parental cell lines. The expression of TPD52L2 was found to be upregulated in OXA-resistant cells. Knockdown of TPD52L2 suppressed cell colony-forming potency, cell growth, and development in OXA-resistant cells. TPD52L2 knockdown also enhanced the PARP and caspase-3 cleavage. ER-associated proteins such as PERK, GRP78, CHOP, and IRE1*α* were found to be elevated in TPD52L2 knockdown cells. ER stress might be involved in TPD52L2 knockdown-induced apoptosis in OXA-resistant gastric carcinoma cells.

## 1. Introduction

Gastric carcinoma is considered to be one of the most commonly occurring malignancies in the digestive system. Worldwide, the incidence of gastric carcinoma has been observed to be decreasing, but its mortality rate remains higher than that of other cancer-associated deaths. A high mortality rate of this carcinoma is correlated with the increasing incidence of drug resistance and its bad prognosis [1]. There is an urgent need to extensively reveal the mechanism of continuously increasing drug resistance and investigate effective therapeutic strategies against gastric carcinoma.

Tumor protein D52-like 2 (TPD52L2), also called TPD54, belongs to the family of tumor protein D52 (TPD52) [2]. The elevated expression level of TPD52 has been associated with a variety of human carcinomas such as ovarian carcinoma [3], prostate carcinoma [4], and breast carcinoma [5]. An elevated TPD52 expression was found to be increasing colony formation, cell proliferation, and tumor migration/invasion [6, 7]. Furthermore, it has been reported that TPD52L2 knockout in glioma cells suppresses their growth rate [8]. Similarly, a decreased level of TPD52L2 expression elevates cell invasive ability, while suppressing cell proliferation and drug sensitivity against glioblastoma cancerous cells [9]. However, the TPD52L2 high expression and its correlation with the increasing resistance of chemotherapeutic drugs against gastric carcinoma remain elusive.

It is well known that chemotherapeutic drugs act via induction of ER stress [10, 11]. It is important to note that moderate ER stress can induce cancerous cells' proliferative ability via elevating their capacity for biosynthesis. Although, extreme ER stress induces cellular apoptosis via initiating ER-associated protein, inositol-requiring kinase 1*α* (IRE1*α*), glucose receptor protein 78 (GRP78), CCAAT-enhancer binding protein (CHOP), and protein kinase RNA-like endoplasmic reticulum kinase (PERK). Moreover, GRP78 is considered to be the principal ER stress biomarker. CHOP is an ER-stress-induced apoptosis marker; PERK and IRE1*α* are ER stress sensor proteins [12–16]. Whether the ER stress trigger is related to the downregulation of TPD52L2 expression in OXA-resistant gastric cells remains to be clarified. Herein, this study explored the TPD52L2 expression and its effect on the proliferation, colony formation, apoptosis, and ER stress of OXA-resistant gastric cells.

## 2. Materials and Methods

### 2.1. Cell Lines and Culture

The human gastric carcinoma cell lines MGC-803 and SGC-7901 were provided by ATCC (Rockville, Maryland, USA), followed by seeding in RPMI 1640 medium (Hyclone, Logan, UT, USA), supplemented with BSA (10 percent) and streptomycin and penicillin (100 units per ml). The cultural flasks were then incubated at 37°C in the presence of 5% CO_2_.

### 2.2. Induction of OXA-Resistant Cell Lines

The OXA-resistant gastric carcinoma cells were induced as reported previously [17]. MGC-803 and SGC-7901 parental cells were trypsinized at the exponential phase, followed by culturing with a 2 nM minimum concentration of OXA (oxaliplatin, Sigma Aldrich, USA). The OXA concentration was gradually elevated (i.e., 0.2 *μ*M) each month. Finally, cell culture was maintained at a 5 *μ*M concentration of OXA. Next, the OXA-resistant cells were cultured without OXA for seven days before the follow-up experiment.

### 2.3. Plasmid Construction and Transfection

Negative-control siRNA (siCon) and three other TPD52L2-targeting siRNAs, i.e., siRNA-1, siRNA-2, and siRNA-3, were specifically generated by GenePharma Company (Shanghai, China). The TPD52L2 siRNA contains the following nucleotide sequences: siRNA-1, 5′-GCGGAGGGTTTGAAAGAATAT-3′, siRNA-2, 5′-GACCATAAAGTCTAAGGTTGT-3′, and siRNA-3, 5′-CTTGGAGACATGAGGAACTCT-3′ besides siCon, 5′-CAGAAGGCAGGCCTCGATAT-3′. Lipofectamine 2000 (Invitrogen, USA) was employed for the transfection of TPD52L2 siRNAs or siCon, following the guidelines provided by the manufacturer.

### 2.4. Cell Viability Assay

Using a 96-well plate, cell lines were incubated with the indicated concentration of OXA at a density of 3 × 10^3^/100 *μ*L for 48 h. Then, MTT dye (5 g/L; Sigma) was added to cells, followed by incubation at 37°C for 4 h, and then, DMSO (150 *μ*L) was mixed into each plate well. Next, the cell and other contents of each well were homogenized for 10 minutes. The O.D. of the mixture was recorded via a microplate reader (Victor2, Wallac, Finland) at 400 nm. The sensitivity of OXA was determined in terms of IC_50_ values. The calculation of IC_50_ values was carried out via GraphPad Prism, VER 5.0 (GraphPad, San Diego, USA).

### 2.5. qRT-PCR Analysis

The TRIzol reagent (Ambion, Foster City, CA)-based total RNA extraction from cells was carried out, followed by RNA reverse transcription using the Primer-Script one-step RT-PCR kit (TaKaRa, Dalian, China). Next, the SYBR Green-based qRT-PCR analysis was carried out using the ABI 7500 instrument (Applied Biosystems, Foster City, CA). The sequence of primers was prepared by Sangon Biotech (Shanghai, China), comprisingTPD52L2, forward, 5'-CTCCTGCTGTTGAGGGTCTG-3', reverse, 5'-CATAGGCGCTAGAGACCTGC-3'; GAPDH, forward, 5'-ACCACCAACTGCTTAGCACC-3', reverse, 5'-CCAGTGAGCTTCCCGTTCAG-3'. With the help of the 2^−ΔΔCt^ method, the relative gene expression level was normalized to the expression level of internal control (GAPDH).

### 2.6. Immunoblotting Analysis

The total cellular protein contents were isolated via radioimmunoprecipitation assay (RIPA) lysis buffer (Beyotime, Shanghai, China). Next, the proteins were analyzed using the BCA Protein Assay Reagent Kit (Beyotime) and were subsequently transferred onto SDS-PAGE, followed by moving to the PVDF membrane (Millipore, USA). Next, the blockage of the PVDF membrane was carried out using primary antibodies (TPD52L2, 1 : 2000; cleaved PARP, 1 : 2000; cleaved caspase-3, 1 : 2000; GRP78, 1 : 2000; PERK, 1 : 1000; CHOP, 1 : 1000; and IRE1*α*, 1 : 1000), followed by incubating at 4°C for 24 h. Then, the membranes were reincubated at 25°C with goat anti-rabbit IgG H&L (HRP) at 1/10000 dilution for 2h. All antibodies were from Abcam (Cambridge, MA). Finally, blot bands were visualized by autoradiography. The protein quantification was carried out via ImageJ software and normalized to GAPDH.

### 2.7. Colony-Formation Assay

For colony-forming assay, SGC-7901 and MGC-803 cells (300 cells per well) from various groups were plated into 6-well plates after infection for 2 days. The medium was changed every three days. After seven days, paraformaldehyde (4%) was used for fixing cells for 0.5 h at ∼25°C. The staining of the fixed cells was carried out with crystal violet (0.5%) for 10 minutes and then photographed and calculated.

### 2.8. Statistical Analysis

All statistical analyses were conducted via GraphPad Prism, VER 5.0 software. Student's *t*-test was employed for comparing the variations between groups, and the data obtained from three independent experiments were represented as mean ± SD. A *p* value <0.05 was considered statistically significant.

## 3. Results

### 3.1. Upregulation of TPD52L2 in Gastric Carcinoma Cells Resistant to OXA

**Figure 1:**
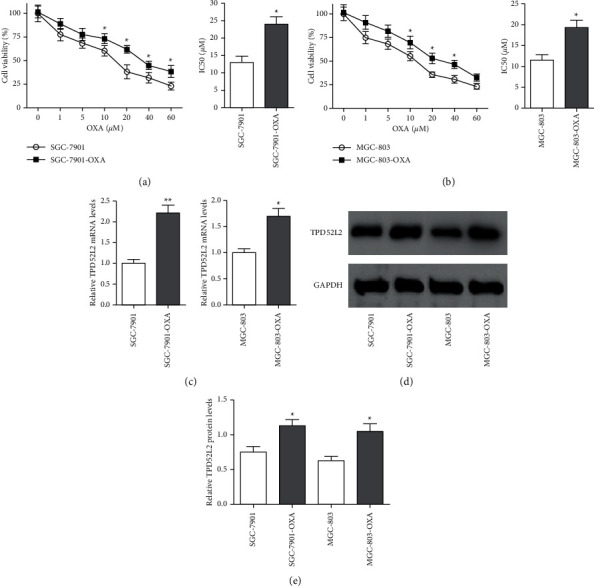
Depiction of the expression of TPD52L2 in OXA-resistant gastric carcinoma cell lines. (a, b) MTT assay was employed for the evaluation of cell viability in cells, i.e., SGC-7901 and MGC-803 cells, and their OXA-resistant counterparts, i.e., SGC-7901-OXA and MGC-803-OXA, after culturing for two days with increasing concentrations of OXA. IC_50_ (*μ*M) values of cells were examined. (c) The mRNA levels of TPD52L2 were identified via qRT-PCR and normalized to the levels of GAPDH that are expressed as fold-change relative to the parental cell lines. (d) The protein expression level of TPD52L2 was determined via immunoblotting with GAPDH as the loading control. (e) Quantification of TPD52L2 protein levels normalized to GAPDH. All values are indicated as mean ± SD of three replicates. ^*∗*^*p* value <0.05 and ^*∗∗*^*p* value <0.01 compared with the corresponding control cells.

The gastric carcinoma cells, i.e., MGC-803 and SGC-7901, were cultured in OXA increasing concentrations for the generation of the OXA-resistant cell lines (MGC-803-OXA and SGC-7901-OXA). The MTT assay was employed for the determination of the cell viability in OXA-resistant cells as well as parental cells. The results revealed that the OXA-resistant cells showed more resistance to OXA, as compared with the MGC-803 and SGC-7901 cell lines. The IC_50_ values for MGC-803-OXA cells and SGC-7901-OXA cells were calculated two days after treatment and were 19.35 *μ*M and 24.11 *μ*M, respectively, and were 1.68 and 1.84 times higher than the IC50 values for MGC-803 and SGC-7901, as shown in Figures 1(a) and 1(b). qRT-PCR and immunoblotting were conducted for the measurement of TPD52L2 expression level in gastric carcinoma cells resistant to OXA. The transcriptional and translational level of TPD52L2 were overexpressed in OXA-resistant gastric carcinoma cell lines, i.e., SGC-7901-OXA as well as MGC-803-OX, as compared with their parental cell lines, i.e., SGC-7901 as well as MGC-803, as depicted in Figures 1(c)–1(e). The results demonstrated TPD52L2 upregulation in gastric carcinoma cells (resistant to OXA) which suggests that TPD52L2 has a potential role in OXA resistance chemotherapy in gastric carcinoma.

### 3.2. Inhibition of OXA Resistance and Colony Formation of Gastric Carcinoma Cells via TPD52L2 Knockdown

**Figure 2:**
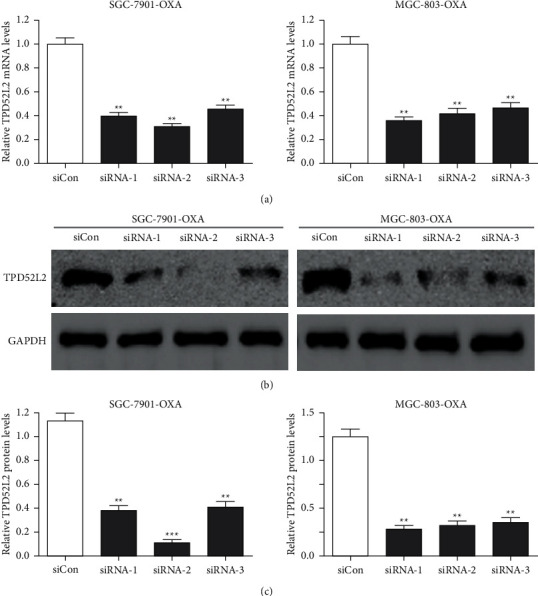
Validation of small interfering (si) RNA-mediated silencing of TPD52L2. The transfection of SGC-7901-OXA and MGC-803-OXA cells with negative-control siRNA (siCon) or three TPD52L2 siRNAs (siRNA-1, siRNA-2, and siRNA-3) for two days. (a) The mRNA expression of TPD52L2 was determined via qRT-PCR, normalized to GAPDH expression, and depicted as fold-change relative to the control siRNA group. (b) The protein expression level of TPD52L2 was determined through immunoblotting with GAPDH as the loading control. (c) Quantification of TPD52L2 protein level normalized to GAPDH. All values are indicated as mean ± SD of three replicates. ^*∗∗*^*p* value <0.01 and ^*∗∗∗*^*p* value <0.001 compared to the siCon group.

**Figure 3:**
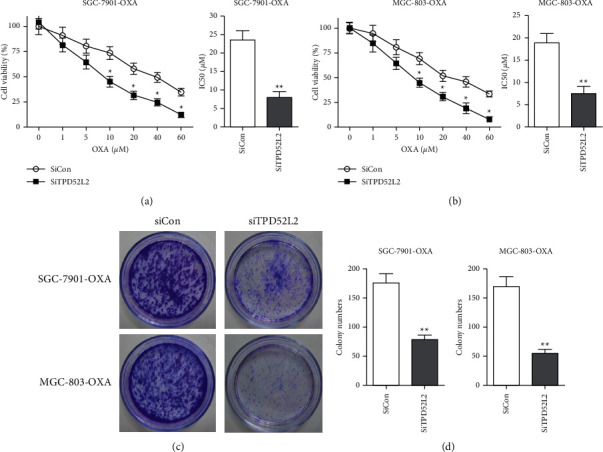
Effect of silencing of TPD52L2 on the viability of cells and colony-forming ability in gastric carcinoma cell lines (OXA resistant). (a, b) Viability of SGC-7901-OXA and MGC-803-OXA cells transfected with siCon or TPD52L2 siRNA-2 (siTPD52L2) was evaluated via MTT assay after culture for two days with increasing concentrations of OXA. IC_50_ (*μ*M) values of cells were measured. (c) Illustrative images of cloning-forming assays. (d) Stained colonies (>1 mm in diameter) were counted. All values are indicated as mean ± SD of three replicates. ^*∗*^*p* value <0.05 and ^*∗∗*^*p* value <0.01 compared to the siCon group.

To analyze the impact of TPD52L2 on the OXA resistance of gastric carcinoma cells, the transfection of OXA-resistant gastric carcinoma cells, i.e., SGC-7901-OXA and MGC-803-OXA, was performed with TPD52L2 siRNAs, i.e., siRNA-1, siRNA-2, and siRNA-3. The results of qRT-PCR revealed that the expression level of TPD52L2 was considerably lowered in gastric carcinoma cells (OXA resistant) when transfected with specifically produced siRNAs, in comparison with cells that were transfected with the negative-control siRNA (siCon), as depicted in Figure 2(a). Immunoblot analysis revealed that TPD52L2 protein also showed considerable downregulation in TPD52L2 knockdown cells, as shown in Figures 2(b) and 2(c). The siRNA-2 (siTPD52L2) was selected for further investigation. In TPD52L2 knockdown transfection, the cell survival and IC_50_ value of OXA were reduced, as compared with the negative-control siRNA (siCon) group, as indicated in Figures 3(a) and 3(b). In the meantime, the TPD52L2 knockdown reduced the number of colonies, as compared to the siCon-transfected cells (Figures 3(c) and 3(d)). Hence, the underlined results suggested that TPD52L2 knockdown blocks OXA resistance and colony formation of gastric carcinoma cells *in vitro*.

### 3.3. TPD52L2 Knockdown Induced the Cellular Apoptotic Process and ER Stress of OXA-Resistant Gastric Carcinoma Cells

**Figure 4:**
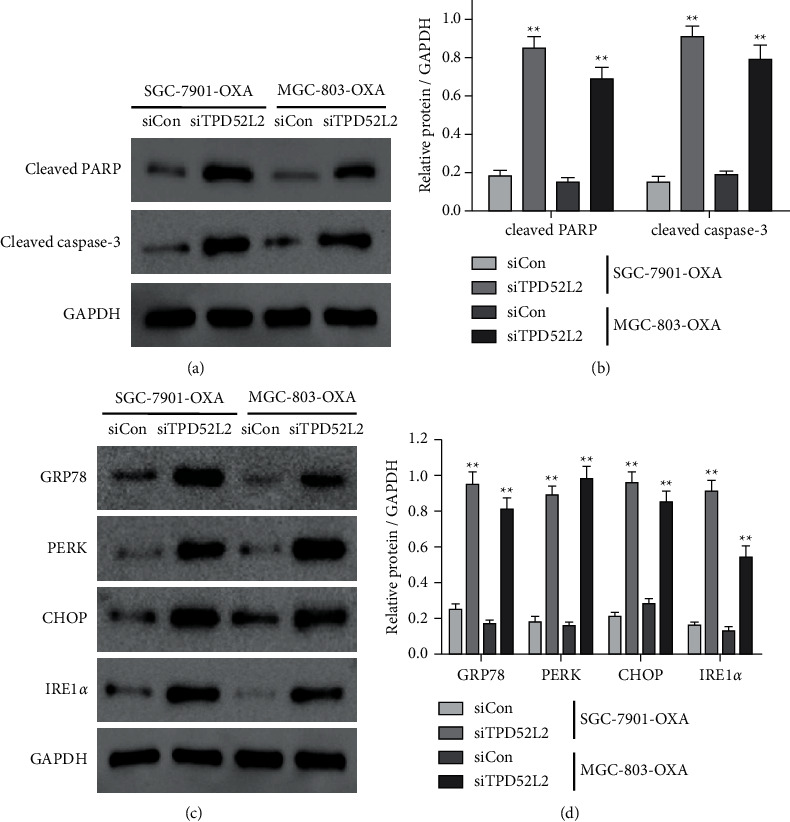
Effect of silencing of TPD52L2 on apoptosis and ER stress-associated proteins in OXA-resistant gastric carcinoma cell lines. (a) Protein expression levels of apoptosis-associated factors, i.e., cleaved PARP and cleaved caspase-3, in SGC-7901-OXA and MGC-803-OXA cells transfected with siCon or siTPD52L2 determined via immunoblotting. (b) The translational levels of apoptosis-associated factors were normalized to GAPDH. (c) Immunoblotting analysis of ER stress-associated proteins, i.e., GRP78, PERK, CHOP, and IRE1*α*. (d) Protein expression levels of ER stress-related factors were normalized to GAPDH. All values are shown as mean ± SD of three replicates. ^*∗∗*^*p* value <0.01 compared to the siCon group of each cell line.

To evaluate the effect of TPD52L2 knockdown on the apoptotic process and ER stress, the expression level of apoptosis-associated factors (cleaved PARP and cleaved caspase-3) and ER stress markers (GRP78, PERK, CHOP, and IRE1*α*) were identified in SGC-7901-OXA and MGC-803-OXA cells transfected with siTPD52L2 in comparison to siCon-transfected cells via immunoblot. The results demonstrated that the TPD52L2 knockdown showed an elevation in the cleaved PARP and cleaved caspase-3 expression, as depicted in Figures 4(a) and 4(b). Moreover, GRP78, PERK, CHOP, and IRE1*α* were considerably elevated with siRNA‐mediated TPD52L2 knockdown (Figures 4(c) and 4(d)). It is well known that the increasing level of GRP78, PERK, and IRE1*α* revealed the occurrence of ER stress and CHOP induction which lead to apoptosis. Together, these findings suggest that TPD52L2 knockdown in SGC-7901-OXA and MGC-803-OXA cells cause cell death that might be correlated with the inhibition of gastric carcinoma cell proliferation (OXA resistant).

## 4. Discussion

OXA is the most common chemotherapeutic agent [18], and chemotherapy is the leading approach for treating advanced stages of gastric carcinoma and adjuvant surgery for gastric carcinoma, although chemotherapy mainly fails due to multidrug resistance [17, 19]. TPD52L2 is a protein that contributes significantly to tumorigenesis and various kinds of carcinomas [20–22]. But, its molecular mechanism regarding its oncogenic role is still a mystery. Our earlier *in vitro* studies have shown that the silencing of TPD52L2 expression inhibited the growth and development of gastric carcinoma cells and colony formation [23]. In the existing study, we identified that TPD52L2 contributes to the resistance of gastric carcinoma cells to OXA and explores the significant molecular mechanism in the regulation of chemoresistance.

In this study, the OXA-resistant human gastric carcinoma cell lines, i.e., SGC-7901-OXA and MGC-803-OXA, were well established via their treatment with an increasing concentration of drug in a continuous and stepwise manner. In comparison with the parental cells, i.e., SGC-7901 and MGC-803, the drug-resistant cells showed a high level of proliferation and IC_50_ value to OXA, which suggests that the cell lines, i.e., SGC-7901-OXA and MGC-803-OXA, are the reliable models of drug resistance for studying the mechanisms underlying drug resistance. In the meantime, we investigated the TPD52L2 upregulation in OXA-resistant gastric carcinoma cells, as compared with OXA-sensitive parental cells which suggest its oncogenic role in gastric carcinoma OXA chemoresistance.

TPD52L2 is a member of the TPD52 family that has a key role in mediating cell proliferation, apoptotic process, and vehicle trafficking [2]. Ren et al.'s study [24] revealed that the elevated expression level of TPD52L2 is correlated with the clinical development and bad prognosis in patients with prostate carcinoma. Furthermore, the TPD52L2 expression inhibition inhibited cell proliferation, migration, and invasion in glioma cell lines [25]. On the contrary, Kato et al. [26] reported that TPD52L2 knockdown improved the number of colonies that are formed in oral squamous cell carcinoma cells. Moreover, the elevated expression level of TPD52 noticeably inhibited the proliferation, migration, and invasion of renal cell carcinoma cells, as well as decreased tumor progression in renal carcinoma xenografts [20]. In this study, the TPD52L2 role was evaluated in gastric cancer cells (OXA-resistant) through the construction of siRNAs to knockdown the expression level of TPD52L2 in cells, i.e., SGC-7901-OXA and MGC-803-OXA, and it was investigated that TPD52L2 silencing leads to the significant decrease in the proliferative and colony-forming capacity of cells. The caspase-3 and PARP-1 activation is a crucial process for the activation of the apoptotic process [27, 28]. We identified that the induction in caspase-3 and PARP-1 activation via TPD52L2 silencing might be a reason for the apoptotic-induced antitumor effect in gastric carcinoma cells (OXA resistant).

It has been indicated that stimulation of ER stress can increase the apoptotic process and reduce the proliferation of gastric carcinoma cells [29]. In the existing study, we found the elevated expression level of PERK, GRP78, CHOP, and IRE1*α* in TPD52L2 knockdown OXA-resistant gastric carcinoma cells, as compared with the control siRNA-transfected cells. Under physiological conditions, the ER stress sensor proteins, including IRE1, PERK, and ATF6, bind with the chaperone (GRP78), which inhibits their activity. When ER stress is activated, an elevated level of GRP78 has been dissociated from IRE1, PERK, and ATF6 and interacts with the misfolded or unfolded protein that results in the initiation of the apoptotic process via CHOP-dependent cascade [30–32]. Hence, ER stress signals result in the triggering of CHOP that might cause an apoptotic-induced antitumor effect in TPD52L2 silencing OXA-resistant gastric cancer cells.

## 5. Conclusions

In summary, we revealed that the elevated expression level of TPD52L2 in OXA-resistant gastric carcinoma cells and TPD52L2 knockdown reduced the viability of cells and colony-forming ability in OXA-resistant gastric carcinoma cells. The apoptosis and ER stress-associated proteins were upregulated through the silencing of TPD52L2. The results suggested that TPD52L2 may cause the resistance of gastric carcinoma cells to chemotherapy and propose a new therapeutic target for treating gastric carcinoma cells. However, the study lacks the observation of TPD52L2 overexpression affecting the biological functions of OXA-resistant cells, which needs to be paid attention to in future studies. Further studies are also needed for the investigation of the antitumorigenic effects of TPD52L2 silencing in animal models.

## Data Availability

Data are available from the corresponding author upon request.
